# Substance P Regulates Environmental Tobacco Smoke-Enhanced Tracheal Smooth Muscle Responsiveness in Mice

**DOI:** 10.1155/2012/423612

**Published:** 2012-08-13

**Authors:** Lan Xiao, Zhong-Xin Wu

**Affiliations:** Department of Neurobiology and Anatomy, Robert C. Byrd Health Sciences Center, West Virginia University, P.O. Box 9128, Morgantown, WV 26506, USA

## Abstract

Environmental tobacco smoke (ETS) is an environmental trigger that leads to airway inflammation and airway hyperresponsiveness (AHR) in susceptible individuals and animals, but the underlying mechanism is not fully understood. Substance P (SP) release from sensory nerve fibers has been linked to AHR. The present experiments characterize the role of SP in tracheal smooth muscle on ETS-increased airway responses. The mice were exposed to either sidestream tobacco smoke (SS), a surrogate to ETS, or filtered air (FA) for 1 day or 5 consecutive days. Contractions of tracheal smooth muscle to SP and electrical field stimulation (EFS) were not significantly altered in 1 of day SS-exposed mice. However, 5 of days SS exposure significantly increased airway smooth muscle contractions to SP and EFS. Administration of CP-99994, an antagonist of the neurokinin (NK)1 receptor, attenuates the SS exposure-enhanced tracheal smooth muscle responses to EFS. Furthermore, the immunohistochemistry showed that SP nerve fibers were increased in tracheal smooth muscle after 5 of days SS exposure. These results suggest that the increased SP production may contribute to SS-enhanced smooth muscle responsiveness in mice trachea.

## 1. Introduction

Environmental tobacco smoke (ETS) is an environmental trigger that leads to airway inflammation and airway hyperresponsiveness (AHR) in susceptible individuals and animals [1–5]. Epidemiological studies show that the probability of developing or exacerbating asthma increases in ETS exposure patients [3–10]. But the underlying mechanism initiated by ETS exposures that affect lung function remains undefined.

The nervous system, including the nerves supplying the airways, is highly susceptible to environmental influences [11]. The airways are innervated through autonomic and sensory nerve fibers [12, 13]. Sensory innervation in the airways is important in the pathogenesis of inflammation associated with asthma. Substance P (SP), a member of the tachykinin family, releases from sensory nerve fibers and has potent effects on airway smooth muscle tone, vascular permeability to protein and mucus secretion [14, 15]. Recent studies have shown that SP plays an important role in antigen- and irritant-induced AHR [2, 16, 17]. Furthermore, the studies including ours found that increased SP in the airway is involved in cigarette smoke exposure-induced AHR and airway inflammation [2, 18–25]. However, the role of SP in cigarette smoke exposure-enhanced airway responsiveness is not clear. 

**Figure 1:**
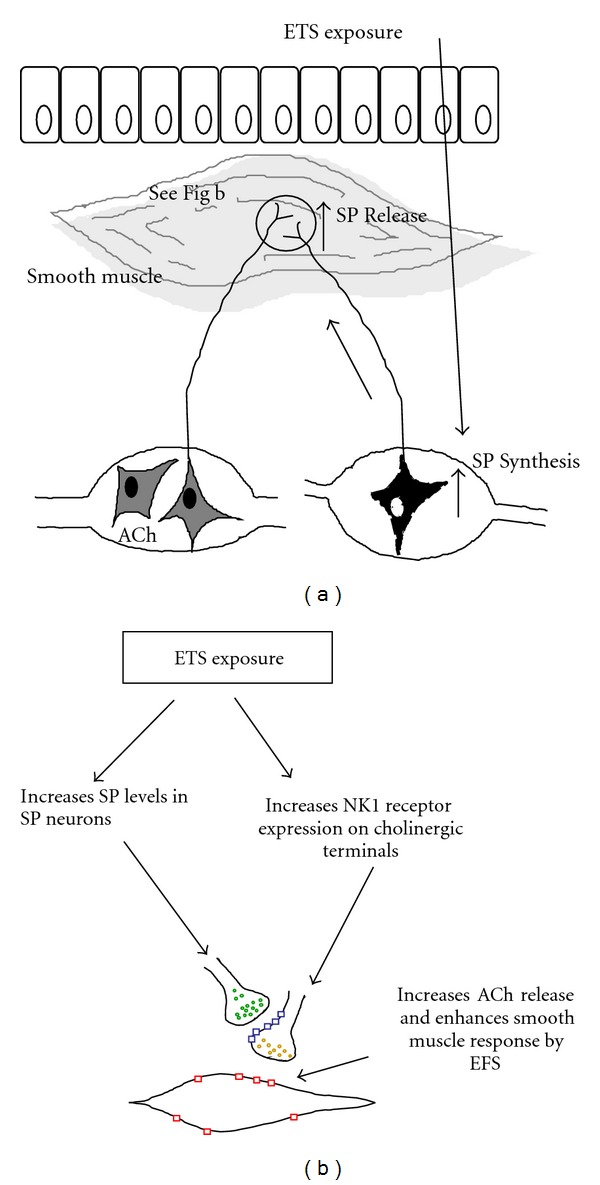
The diagrams demonstrate the effect of increasing SP and NK1 receptor expression on cholinergic nerve of airway smooth muscle. The airway smooth muscles are innervated by cholinergic and sensory nerve fibers (a). The enhancing SP associates with NK1 receptor on the surface of cholinergic neurons, which alters the sensitivity of cholinergic neurons or activates cholinergic neurons and facilitates ACh release from cholinergic nerve (b).

Recent studies found that SP also acts as a neuromodulator increasing the tracheal smooth muscle contractions to electrical field stimulation (EFS) [26–30]. Both of Watson and Hall's studies found that exogenous and endogenous SP facilitated EFS-induced tracheal contractions, but had no effect on contractions induced exogenous ACh [26, 27]. Nadel's group further showed that SP potentiated airway smooth contraction to EFS in a concentration-dependent fashion [28, 29]. Tournoy et al. also found that EFS-induced airway smooth muscle contraction was decreased in NK1 receptor knockout mice compared with wild-type control [30]. All of these studies suggest that SP enhances EFS-induced airway smooth contraction due to increasing the release of ACh (Figure 1). Thus, we hypothesized that ETS exposure increases SP from sensory nerves or changes NK receptor, which facilitates ACh release from cholinergic nerve and enhances airway smooth muscle responses (Figure 1). The enhanced airway smooth muscle response may contribute to increased susceptibility to AHR. The present experiments characterize the role of SP on ETS-enhanced airway smooth muscle responses. 

## 2. Material and Methods 

ICR mice (Harlan, Indianapolis, IN, USA) were housed with access to food and water ad libitum in an FDA-approved facility. All procedures were performed in accordance with the recommendations of the *Guide for the Care and Use of Laboratory Animals*, published by the National Institutes of Health, and the protocols were approved by the WVU Animal Care and Use Committee no. 05-0503. The animals were treated humanely and with regard for alleviation of suffering.

### 2.1. SS Exposure

By classical definition, environmental tobacco smoke (ETS) is a diluted mixture of the smoke given off by the burning end of a tobacco product (side stream smoke, ~85%) and the smoke exhaled by smokers (mainstream smoke, ~15%). Based on previous ETS exposure studies [1, 31, 32], we used sidestream tobacco smoke (SS) as a surrogate to ETS.

The two-month-older mice were exposed to either SS or filtered air (FA) for 6 h/day for 1 day or 5 consecutive days. The trachea and lung tissues were collected 16 h after SS or FA exposure. A major goal of the current study was to understand possible neuronal mechanisms by SS that affect lung function. The SS exposure protocol and methods used in this study have been described in our recent publication [1]. Briefly, mice were randomly placed in an exposure chamber (BioClean, DuoFlo model H 5500, Lab Products Inc) that measured 1.92 × 1.92 × 0.97 m (3.58 m^3^). The mice were housed in separate cages located in the exposure chamber. Sidestream smoke from Marlboro filter cigarettes (Phillip Morris, Richmond, VA, USA) was introduced into the exposure chamber at a rate of four cigarettes every 15 minutes for 6 hours using a smoking machine (RM 1/G, Heinr Borgwaldt GmbH, Hamburg, Germany). At the end of the 6 h exposure period, the exhaust fan unit was turned on to rapidly lower the level of smoke in the exposure chamber. The mice were then transported to the animal facilities overnight. The concentrations of carbon monoxide in the exposure chamber were monitored and kept to an average of about 50 parts per million (ppm), relative humidity was about 50%, and temperature was about 23°C. Total suspended particulate concentration was about 1.1 mg/m^3^, similar to exposure levels used by others to approximate the cloud of particulates surrounding a person during active smoking [32]. 

### 2.2. Measurement of Tracheal Smooth Muscle Contraction

Fresh tracheas from mice 16 h after FA or SS exposure were cut into 3 mm wide rings, mounted in holders, and maintained in gassed (95% O_2_-5% CO_2_) modified Krebs-Henseleit solution at 37°C with a composition (in mM) as follows: 113 NaCl, 4.8 KCl, 2.5 CaCl_2_, 1.2 MgSO_4_, 24 NaHCO_3_, 1.2 KH_2_PO_4_, and 5.7 glucose, pH 7.4. Each tracheal ring was loaded into a pair of stainless-steel hooks, suspended in tissue holders. Each holder was anchored in a 10-mL water-jacketed organ bath, and the top steel hook was attached to a force-displacement transducer connected to a recorder (Gould Instruments, Valley View, OH, USA). The rings were equilibrated for 60 min at a resting tension of 0.25 g, determined by preliminary studies, during which time the modified Krebs-Henseleit solution in the baths was changed every 15 min. After equilibration, methacholine (MCh) responses were constructed by adding 10^−5^ M MCh to the bath and SP cumulative concentration-response curves were constructed by adding a series of concentrations of SP to the bath in half-log-increment concentrations ranging from 10^−9^ to 10^−5^ M. After concentration-response curves were completed, electrical-field-stimulation- (EFS-) induced responses were obtained with a stimulator (model S48, Grass Instruments, Richmond, VA, USA). Frequency-response curves were constructed by increasing the frequency from 0.3 to 30 Hz using a submaximum voltage of 120 V, 0.2-ms pulse duration, and 10 s train duration. Between each stimulation period, 10 min were allowed for the previous response to return to baseline. EFS-induced contractions were normalized by the percent response of each tissue to 10^−5^ M MCh. In some experiments, atropine (10^−6^ M) was added to Krebs solution to verify that the responses elicited by EFS were mediated by the release of ACh. To determine the possible involvement of endogenously released SP, some experiments were given CP-99994 (final concentration 10^−6^ M), SP receptor antagonist, which was incubated at least 30 min before addition of SP or EFS. The dose of this antagonist was determined by dose-response curves.

### 2.3. Immunohistochemistry

The procedures for immunohistochemical quantification of airway nerves have been described previously [1, 33, 34]. Briefly, in a separate group of mice-exposed using the same SS exposure protocol, tracheas were removed 16 h after SS or FA exposure, fixed in picric acid-formaldehyde fixative for 3 h, and rinsed three times with a 0.1 M phosphate-buffered saline containing 0.15% Triton X-100. The tracheas were dissected and frozen in isopentane, cooled with liquid nitrogen, and stored in airtight bags at −80°C. The tracheas were oriented with the dorsal surface uppermost so the tracheal muscle would be sectioned in a coronal plane. 

Cryostat sections (12 *μ*m thick) were collected on gelatin-coated coverslips and dried at room temperature. The cryostat sections on coated coverslips were covered with SP antibody diluted 1 : 100, incubated at 4°C for overnight, and rinsed with a 1% bovine serum albumin + phosphate-buffered saline containing 0.15% Triton X-100 three times, with 5 min being allowed for each rinse. The sections were then covered with fluorescein isothiocyanate-labeled goat antirabbit antibody diluted 1 : 100, incubated at 37°C for 45 min, and rinsed. After all immunohistochemical procedures were conducted, the coverslips were mounted with Fluoromount and observed with a fluorescence microscope equipped with fluorescein (excitation wavelengths 455–500 nm, emission wavelengths > 510 nm). Controls consisted of testing the specificity of primary antiserum by absorption with 1 *μ*g/ml of the specific antigen. Nonspecific background labeling was determined by omission of primary antiserum. 

For measuring nerve fiber density in tracheal smooth muscle, we collected images of control, SP nerve fibers in series under the Zeiss LSM 510 confocal microscope. A series of images representing all of the tracheal smooth muscles in a section was collected in digital files and saved to an internal database and measured using Optimus software. We selected regions of smooth muscle using the rhodamine channel to avoid possible bias created by the presence or absence of nerve fibers. The smooth muscle regions were outlined to measure total cross-sectional area of smooth muscle. The microscope was then switched to reveal nerve fibers in the fluorescein channel, and the image was digitally captured. The threshold levels were manually adjusted to subjectively optimize the appearance of fluorescent nerves. The area of nerve fibers was determined by segmentation with the Optimus software. Then nerve fiber area was standardized to the total cross-sectional area of smooth muscle. The final value of nerve fiber density is expressed as % of dividing the SP nerve fiber area by the total area of smooth muscle. At least 10 measurements were made for each section, and 15 sections were measured in each animal.

### 2.4. SP Enzyme-Linked Immunosorbent Assay

 SP ELISA assay in lung tissue was conducted as in our previously described work [1]. Lungs were obtained from each animal 16 hours after SS exposure. The specimens were weighed, homogenized, and centrifuged (40,000 g). Supernatant fractions were collected, filtered, and frozen at −80°C until assay. The concentration of SP (39–2500 pg/mL) in each sample was assayed using the SP immunoassay system (R&D systems, Minneapolis, MN, USA) according to manufacturer's instructions. All samples were running in duplicate.

### 2.5. Data Analysis

Unless otherwise stated, values are means ± SE. Contractions elicited by EFS and SP are expressed as a percentage of the contraction elicited by MCh 10^−5^ M. The half-maximal effective concentration (EC_50_) values for SP were calculated using a four-parameter logistic curve fit (Sigmoidal, SigmaPlot 2000) and were presented with 95% confidence intervals. Force development was expressed by normalizing force (g) divided by the wet weight of the tissue. Nerve fiber density was expressed as % area of SP-immunoreactive nerve fibers in the total area of the smooth muscle. Statistical analyses of immunohistochemistry and EC_50_ were performed using Student's *t*-test. Statistical analysis of EFS was performed using two-way repeated-measures analysis of variance. One factor between the groups was SS exposure; the other factor within the group was EFS or SP effect. When the main effect was considered significant at *P* < 0.05, pairwise comparisons were made with a post hoc analysis (Fisher's least significant difference). *P* < 0.05 was considered significant, and *n* represented the number of animals studied. 

### 2.6. Materials

MCh chloride and atropine sulfate were obtained from Sigma Chemical (St. Louis, MO, USA). CP-99994 was obtained from Pfizer (Groton, CT, USA). SP and SP antibody were obtained from Peninsula (Belmont, CA, USA). Fluorescein isothiocyanate-labeled goat antirabbit antibody was obtained from ICN Immunobiologicals (Costa Mesa, CA, USA). 

## 3. Results

### 3.1. Effect of SS on Airway Responsiveness

**Figure 2:**
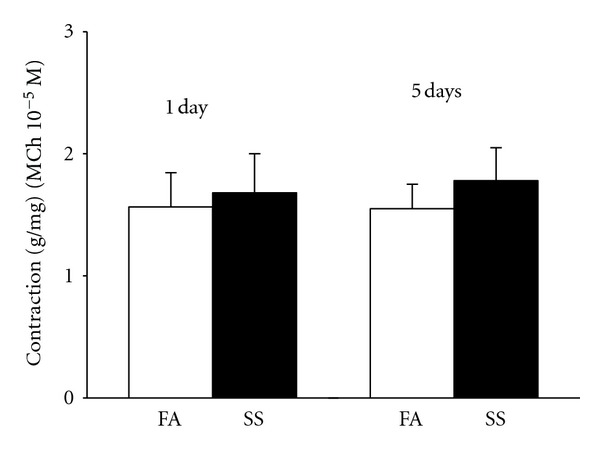
Tracheal smooth muscle contraction was generated by 10^−5^ M methacholine (MCh) in 1 day (*n* = 16 in each group) and 5 days (*n* = 30 in each group) SS-(solid bars) or FA-(open bars) exposed tracheal rings. Values are means ± SE.

The initial experiments were intended to find if SS exposure increases airway smooth muscle contraction to MCh. There was no significant difference in the tracheal smooth muscle contraction to MCh (10^−5^ M) between 1 day-SS-exposed (*n* = 16) mice and FA exposed mice (*n* = 16) (Figure 2). Although 5 days of SS exposure slightly increased smooth muscle contraction to MCh, there was no significant difference in airway responses to MCh between 5 days of SS-exposed (*n* = 30) mice and FA exposed mice (*n* = 30) (Figure 2). 

**Figure 3:**
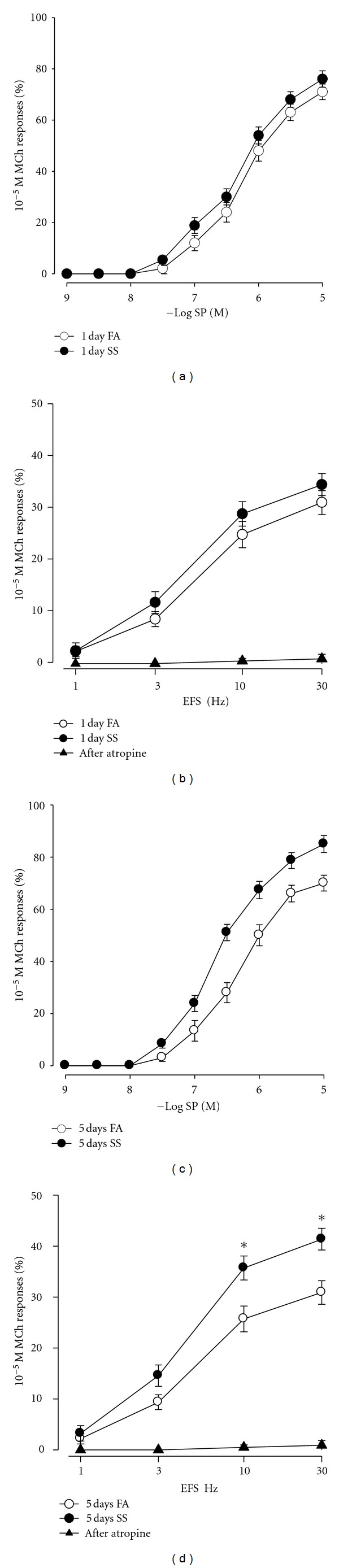
Cumulative concentration-response curves for SP (a, c) and frequency-response curves for electrical field stimulation (EFS; b, d) in tracheal smooth muscle after 1 day (a, b) and 5 days (c, d) exposure to FA (open circles) or SS (circles). The solid triangle showed that atropine (final concentration 10^−6^ M) totally abolished EFS-induced airway smooth muscle contraction. Responses to SP and EFS are plotted as a percentage of the 10^−5^ M MCh response. Values are means ± SE; *n* = 16 in each group. *Significant difference in EFS between FA and SS exposure, *P* ≤ 0.05.

The next experiments were intended to figure out the effect of SS on airway responsiveness to SP and EFS. The cumulative dose-response curve for SP was not significantly shifted to the left after 1 day of SS exposure (Figure 3(a)). There is no significant difference between EC_50_ values in SS exposure (6.17 ± 0.11) and FA exposure (5.96 ± 0.14). Also the frequency-response curve to EFS was not significantly altered 1 day of SS exposure (Figure 3(b)). However, the cumulative dose-response curve for SP was markedly shifted to the left after 5 days exposure to SS (Figure 3(c)), there is significant difference between EC_50_ values in SS exposure (6.65 ± 0.09) and FA exposure (6.01 ± 0.11). Also, a leftward shift in the frequency-response curve to EFS was observed after 5 days of SS exposure (Figure 3(d)), and contractions produced by EFS at 10 Hz and 30 Hz were significantly increased after 5 days of SS exposure. The pretreatment with atropine (final concentration 10^−6^ M) totally abolished EFS-induced airway smooth muscle contraction in both FA and SS-exposed mice (Figures 3(b) and 3(d)), indicating that EFS-induced airway smooth muscle contraction mainly involves endogenously acetylcholine (ACh) release. 

### 3.2. Effects of NK1 Antagonist on 5 Days of SS-Enhanced Airway Responsiveness

**Figure 4:**
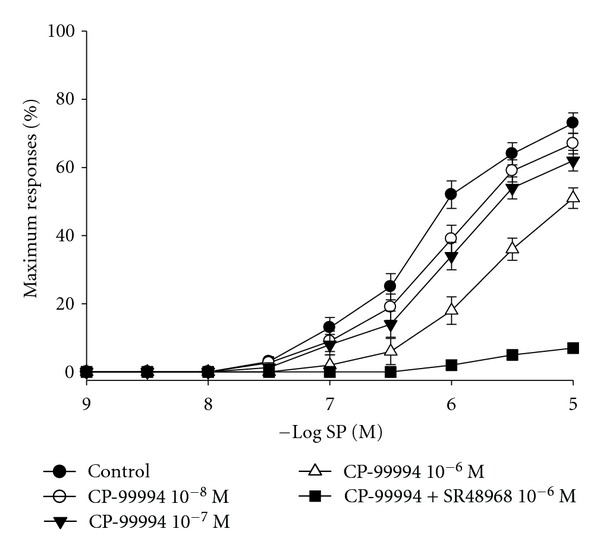
The effect of different concentrations of CP-99994 on cumulative concentration-response curves for SP in isolated mice tracheal smooth muscle. Values are means ± SE, *n* = 6 in each group.

To ensure sufficient reduction of SP effects, the effective dosage of NK1 receptors antagonist, CP-99994, was tested first. In separate experiments, the different concentrations of CP-99994 were tested by SP cumulative doses (10^−9^ M to 10^−5^ M). The SP cumulative dose-response curves were significantly shifted to the right in a concentration-dependent manner by CP-99994 (Figure 4). EC_50_ values for SP are 6.04 ± 0.14 (control), 5.83 ± 0.12 (CP-99994 10^−8^ M), 5.64 ± 0.11 (CP-99994 10^−7^ M), and 5.56 ± 0.11 (CP-99994 10^−6^ M). There is significant difference in EC_5_ between control and CP-99994 10^−6^ M. The combination of CP-99994 (10^−6^ M) and NK2 receptors antagonist SR48968 (10^−6^ M) virtually abolished SP-induced airway smooth muscle contraction, indicating that CP-99994 10^−6^ M is effective dose to block SP-induced airway smooth muscle contraction. Thus, next experiment, CP-99994 10^−6^ M was chosen to test the role of SP on 5 of days SS-enhanced airway smooth muscle responses.

**Figure 5:**
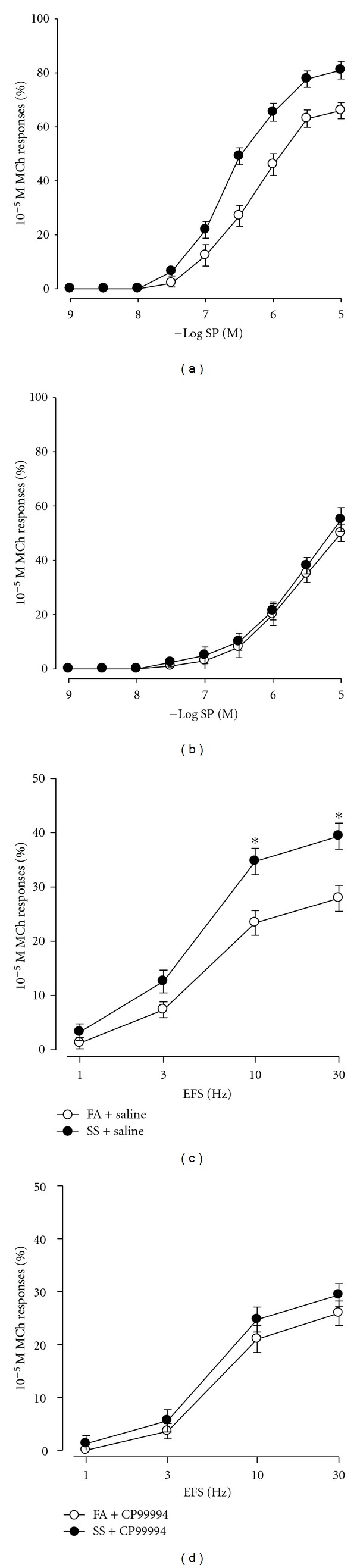
The effect of saline (a and c) or CP-99994 (b and d) on cumulative concentration-response curves for SP and frequency-response curves for EFS in isolated mice tracheal smooth muscle after exposure to 5 days FA (open circles) or SS-(solid circles). Values are means ± SE; *n* = 14 in each group. *Significant difference in EFS between FA and SS exposure, *P* ≤ 0.05.

CP-99994 (final concentration 10^−6^ M) was incubated at least 30 min before using SP or EFS. Cumulative concentration–response curve for SP and the EFS-stimulated contractions in saline pretreated groups after 5 of days SS exposure (Figures 5(a) and 5(c)) demonstrated similar changes as those described above, 5 days of SS exposure. The contractions produced by EFS at 10 Hz and 30 Hz were significantly increased in saline pretreated groups after 5 days of SS exposure. CP-99994 did not significantly affect EFS-induced contraction in FA-exposed animals. The tracheal smooth muscle contractions to EFS at 10 and 30 Hz were not significantly altered in the CP-99994 treatment group (10 Hz: 21.44 ± 2.54%; 30 Hz: 25.8 g ± 2.31%) compared with the saline group (10 Hz: 23.36 ± 2.28%; 30 Hz: 27.89 ± 2.29%) in FA exposed mice. However, CP-99994 significantly inhibited the SS-enhanced smooth muscle responses to EFS at 10 and 30 Hz (Figure 5(d)). There are no significant differences in EFS-induced tracheal smooth muscle contractions between 5 of days SS-exposed mice and FA exposed mice after CP-99994 treatment. Also, the enhanced airway responses to SP were total abolished by CP-99994 treatment (Figure 5(b)). 

### 3.3. Effect of SS on Changes of SP in Trachea and Lung

**Figure 6:**
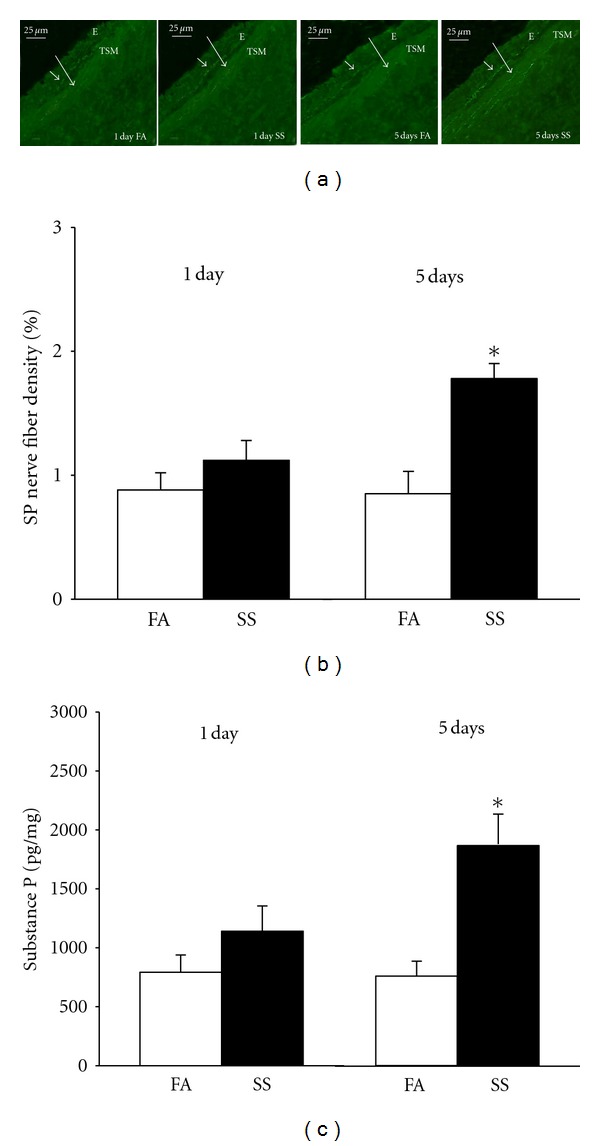
The effect of SS on SP in tracheal smooth muscle and lung. (a) The fluorescence photomicrographs of SP nerve fibers in tracheal epithelium (E) and smooth muscle (TSM) in 1 day and 5 days of FA or SS-exposed mice. Arrows: the localization of SP immunoreactive nerve fibers. (b) The changes of SP nerve fiber density in tracheal smooth muscle after FA (opened bar) or SS (closed bar) exposure. (c) The changes of SP in lung after FA (opened bar) or SS (closed bar) exposure measured by ELISA. Values are means ± SE; *n* = 6 in each group. *Significant difference comparing corresponding data between FA and SS animals, *P* ≤ 0.05.

SP nerve fibers in the tracheal smooth muscle of mice were analyzed based on the immunohistochemical localization by fluorescein. The SP nerve fibers were mainly localized on tracheal smooth muscle and epithelium (Figure 6(a)). There is no significant difference in density of SP nerve fibers in the airway smooth muscle between 1 day of SS exposure and FA exposure (Figures 6(a) and 6(b)). However, the density of SP nerve fibers in the airway smooth muscle was significantly increased after 5 days of SS exposure (Figures 6(a) and 6(b)). These findings suggest that 5 days of SS exposure increases SP content in tracheal smooth muscle. Furthermore, the SP level in lung was measured using ELISA. ELISA data showed that SP levels in lung were significantly elevated in 5 days of SS-exposed mice compared with FA exposure (Figure 6(c)). 

## 4. Discussion

The results obtained from the current study show that exposure to SS significantly enhances tracheal smooth muscle responsiveness in the mice, as evidenced by elevated contractility to SP and EFS. The elevation of airway smooth muscle responses by SS exposure was attenuated by treatment with a NK1 receptor antagonist, indicating that SP played a key role in the enhancement of smooth muscle contractile responses. Our previous study showed that exposure to irritant changes SP airway innervation and enhances tracheal smooth muscle responsiveness [25, 35], suggesting that SP may contribute to irritant-enhanced smooth muscle responsiveness. The current results from immunohistochemistry found that SS exposure changes SP innervation of tracheal smooth muscle, directly indicating that increased SP level in airway involved SS-enhanced smooth muscle responsiveness.

Airway sensory nerves play a central role in airway regulation [36]. SP localized in the peripheral endings of sensory nerves innervating the lung and airways originates in nerve cell bodies located in sensory ganglia [37, 38]. Stimulation of sensory nerves by inhalation of irritants is known to trigger the release of SP from afferent endings [2, 25, 39, 40]. The data in the present study found that SS-enhanced-airway smooth muscle contractions to EFS were attenuated by SP receptor antagonist, directly indicating that SP is involved. However, the role of SP in smoke exposure-enhanced airway responsiveness is not clear. Recent studies found that SP acts as a neuromodulator altering airway responsiveness to EFS [26–30]. Watson and Hall's studies found that exogenous and endogenous SP facilitated electrical field stimulation (EFS)-induced tracheal contractions, but had no effect on contractions induced exogenous ACh [26, 27]. Nadel's group further showed that SP potentiated airway smooth contraction to EFS in a concentration-dependent fashion [28, 29]. Thus, one possible explanation for our finding is that enhanced SP in airway after SS exposure acted as mediator, which altered airway responsiveness to EFS. Our result found that administration of atropine completely blocks airway smooth muscle responsiveness to EFS in both FA and SS-exposed animals, demonstrating that smooth muscle contraction to EFS in mice trachea is entirely atropine sensitive and totally depends on endogenous ACh release from parasympathetic nerve terminals. It also indicates that there is no SP release in EFS-induced airway responses. Furthermore, our MCh experiments found that SS exposure did not increase airway contractions to MCh and suggested enhanced sensitivity of airway smooth muscle was not involved in SS exposure. Thus the logical explanation is that enhanced SP acts as a neuromodulator increasing the release of acetylcholine (ACh) from parasympathetic nerve. Although the exact mechanism of enhanced ACh release from parasympathetic nerve was not determined in the present study, the finding that the SP receptor antagonist CP-99994 significantly attenuated the effect of SS on EFS responses in trachea suggests the idea that SS exposure induces SP upregulation. The enhancing SP may associate with NK1 receptor on the surface of cholinergic neurons, which alters the sensitivity of cholinergic neurons or activates cholinergic neurons and facilitates ACh release from cholinergic nerve. 

Our data also found that contractions of airway smooth muscle to EFS at 10 and 30 Hz were slightly decreased in the CP-99994 treatment group (10 Hz: 21.44 ± 2.54%; 30 Hz: 25.89 ± 2.31%) compared with the saline treatment group (10 Hz: 23.36 ± 2.28%; 30 Hz: 27.89 ± 2.29%) after FA exposure. It indicates that normal level of SP may involve airway responses to EFS. Tournoy et al. study that EFS-induced airway smooth muscle contraction was decreased in NK1 receptor knockout mice compared with of NK1 receptor wild-type controls [30] supports that normal level of SP in airway smooth muscle involved airway responses to EFS, although there is no direct effect of SP on EFS-induced airway responses.

The present experiment also showed that SP dose responses are enhanced after 5 days SS exposure. One possibility is that sensitivity of airway smooth muscle to SP is enhanced by SS exposure. However, it is unlikely that the sensitivity of airway smooth muscle was enhanced due to sensitivity of airway smooth muscle to MCh that was not significantly changed. Alternatively, an altered density and/or expression of the NK receptors or properties of NK receptors (e.g., affinity) on the airway smooth muscle may be involved by SS exposure. Our experiments used three concentrations of CP 99994 and detected that even high-dose CP 99994 (1 *μ*M) only partially but incomplete inhibited exogenous SP-induced airway contraction, suggesting that mice airway may contain a heterogeneous population of receptors. Furthermore, our experiment uses combination with NK1 and NK2 receptor antagonist (SR 48968) treatment and found that combination with NK1 and NK2 receptor antagonist treatment virtually eliminated the response to SP in airway, indicating both NK1 and NK2 receptors mediate the contractile response to SP. Thus, the possible explanation is that SS exposure alters density and/or expression of the NK receptors or properties of NK receptors (e.g., affinity) on the airway smooth muscle, which affect airway repose to SP. 

The previous studies including ours have found that childhood exposure to ETS is a significant risk factor for exacerbation of asthma, but studies of smoking in adults are less conclusive. There are evidence that mainstream cigarette smoking exposure may decrease the incidence of some chronic inflammatory and have a beneficial effect of smoking on airway responsiveness [41–44]. These studies found that exposure to mainstream cigarette smoke attenuates airway responses to ACh, methacholine [41–43] and neurokinin A [44], also decreased airway inflammation comparing with nonsmoking animals [43]. However, it has also been reported that mainstream cigarette smoking or ETS exposure can enhance allergic airway inflammation [45, 46] and increase airway responses to Ach [45, 47], endothelin [48, 49]. Thus, the relationship between cigarette smoking and airway responsiveness is complex. The different methods and doses of cigarette smoke exposure may induce different airway responsiveness. The low levels of ETS or mainstream cigarette smoke promote allergic sensitization and increase airway responsiveness [46, 50], whereas high levels of mainstream cigarette smoke exposure associated with direct smoking suppress allergic airway inflammation and airway hyperresponsiveness [41–43, 50]. In our present experiment, we used low levels of sidestream tobacco smoke (SS) as a surrogate to ETS instead of mainstream cigarette smoke, and found SS exposure significantly enhances tracheal smooth muscle responsiveness SP and EFS, supporting that low levels of ETS enhance the development of allergic airway inflammation. 

Cigarette smoke exposure activates sensory nerve fibers [23–25]. One of the significant findings in the current results from immunohistochemistry found that SS enhances SP expression in airway nerves. These data provide direct evidence that SP nerve fibers in airway smooth muscles were increased by 5 days of SS exposure. Clinic data demonstrated that SP nerve fiber density was increased in airway smooth muscle of severe asthmatics [51]. A more recent study showed that the SP nerve fiber density was increased in airway epithelium from human subjects with persistent nonproductive cough [52]. These studies show that increased levels of SP in human airway sensory nerves may contribute to alteration of airway responses. 

In conclusion, our results show that 5 days of SS exposure increases SP nerve fibers innervating tracheal smooth muscle. At the same time, smooth muscle responses to EFS and SP are increased in tracheal segments. Administration of CP-99994, an antagonist of the NK1 receptor, attenuates the SS exposure-enhanced tracheal smooth muscle responses to EFS, indicating that the enhanced responses are dependent on SP increase from sensory nerves or change of NK receptors density in the airway smooth muscle. Combining immunohistochemistry finding that SP nerve fibers were increased in tracheal smooth muscle after 5 days of SS exposure, the current study suggests that the increased SP production by SS exposure may contribute SS-enhanced smooth muscle responsiveness in mice trachea.

## Abbreviations

ACh: Acetylcholine
ELISA: Enzyme-linked immunosorbent assay
ETS: Environmental tobacco smoke
FA: Filtered air
MCh: Methacholine
NFD: Nerve fibers density
SP: Substance P
SS: Sidestream tobacco smoke.
